# Body location of “New World” cutaneous leishmaniasis lesions and its impact on the quality of life of patients in Suriname

**DOI:** 10.1371/journal.pntd.0008759

**Published:** 2020-10-23

**Authors:** Ricardo V. P. F. Hu, Sahienshadebie Ramdas, Pythia Nieuwkerk, Ria Reis, Rudy F. M. Lai A Fat, Henry J. C. de Vries, Henk D. F. H. Schallig

**Affiliations:** 1 Dermatology Service, Ministry of Health, Paramaribo, Suriname; 2 Amsterdam University Medical Centers, Academic Medical Centre at the University of Amsterdam, Department of Medical Microbiology and Infection Prevention, Experimental Parasitology Unit, Amsterdam, the Netherlands; 3 Amsterdam Institute for Social Science Research, University of Amsterdam, the Netherlands; 4 Amsterdam University Medical Centers, Academic Medical Centre at the University of Amsterdam, Department of Medical Psychology Amsterdam, the Netherlands; 5 Department of Public Health and Primary Care, Leiden University Medical Centre, the Netherlands; 6 Department of Dermatology, Academic Hospital, Paramaribo, Suriname; 7 Centre for Infection and Immunity Amsterdam (CINIMA), Academic Medical Center, University of Amsterdam, Amsterdam, the Netherlands; 8 Department of Dermatology, Academic Medical Center, University of Amsterdam, Amsterdam, the Netherlands; 9 STI Outpatient Clinic, Cluster Infectious Diseases, Public Health Service Amsterdam, Amsterdam, the Netherlands; KU Leuven, BELGIUM

## Abstract

Cutaneous leishmaniasis (CL) is a chronic skin infection caused by *Leishmania* parasites, causing single or multiple skin nodules and ulcers on the exposed body locations. Healing of lesions is followed by scar formation. Active and healed CL lesions may affect patient’s health related quality of life (HRQL). The aim of this study was to determine whether the body location of the leishmaniasis lesions affects the HRQL of localized CL patients in Suriname. The HRQL of 163 patients with CL was assessed by Skindex-29 and EQ-5D/VAS questionnaires. Forty-six patients out of the total study population also participated in a qualitative anthropological study involving in depth interviews. All patients were allocated in 4 groups in the following hierarchy: head and face, upper limbs, lower limbs and trunk. Patients with lesions on the lower limbs had significantly higher Skindex-29 scores, indicating worse HRQL, in the symptom scale compared to lesions on head/face and trunk. The lower limb group was more likely to report problems in the dimensions self-care, mobility, daily activities and pain/discomfort of the EQ-5D. Little to no social stigma was reported in the in-depth interviews. The findings of this study indicate that Surinamese patients with CL lesions located on the lower limbs had more impairment in HRQL than on other body locations. Stigma related to CL seems to be virtually absent in Suriname.

## Author summary

Cutaneous leishmaniasis (CL) is a parasitic skin infection characterized by one or more skin lesions. Depending on the location of the lesions, patients may experience difficulties in their day-to-day life including stigma. The aim of this study was to evaluate the quality of life of CL patients with lesions. We collected the data among 163 CL patients with two different questionnaires assessing various parts of their quality of life; 46 patients provided additional information through in depth interviews. All patients were divided in four groups according to location of lesions on the body: head and face, upper limbs, lower limbs, and trunk. We found that patients experienced more difficulties when lesions were located on the lower limbs. Little to no social stigma was reported in the in-depth interviews. The study concluded that Surinamese patients with lesions on the lower limbs had a lower quality of life compared to other body locations.

## Introduction

Leishmaniasis is a poverty-related disease with an estimated 0.7 to 1 million new cases reported per year from nearly 100 endemiccountries [1]. Leishmaniasis is caused by different species of the genus *Leishmania*, which are transmitted through the bite of an infected female sand fly of the genus *Phlebotomus* or *Lutzomyia*. Cutaneous leishmaniasis (CL) is the most prevailing clinical manifestation of leishmaniasis. WHO estimated an incidence of 1.5 million cases per year [2]. CL is a chronic infection with a spectrum of clinical presentations; most frequent is (extensive) skin ulceration, which can lead to disfiguring scar formation. The skin ulcers can be single or multiple, and satellite lesions or nodular lymphangitis may be present. In rare cases mucosal involvement can occur. CL is endemic in Suriname at an annual rate of 6/1000, with around 250 cases officially reported each year, and mainly presents as localized cutaneous leishmaniasis, (LCL). The majority of cases is caused by *L*. *(V*.*) guyanensis*, and the disease mainly affects young males involved in mining, logging and tourism operations; leishmaniasis is considered to be an increasing public health problem [3,4].

CL lesions are located on parts of the body exposed to the vector such as face, hands and legs and can be troublesome and unsightly. Studies performed in Afghanistan, Pakistan, Turkey and Iran showed it also has a great social impact on the health-related quality of life (HRQL) as the scarring of patients’ lesions can result in substantial stigma [5,6,7,8]. Stigmatization occurs in all age groups. CL affected children are isolated from others in the family, and women are particularly victimized as they are considered unacceptable for marriage and are separated from their children during the illness and not allowed to breastfeed. Due to the stigma associated with CL lesions and scars, patients can suffer from anxiety and depression [7]. The United Nations office for the coordination of Humanitarian Affairs in Afghanistan (IRIN) reported in 2008 that girls drop out of school when experiencing CL especially in the face. Several men cancelled engagement to fiancées facially disfigured by CL [9]. Few studies, however, have assessed the impact of CL on the HRQL and stigmatization in the New World [10].

The relation between location of skin lesions on the body and the impact on HRQL has been studied in other skin diseases e.g. vitiligo. In the study performed by Silverberg and Silverberg [11] it was shown that different distributions of vitiligo lesions have different effects on HRQL. Sexual dysfunction was associated with genital lesions, whereas self-consciousness was associated with lesions on exposed areas, such as face and limbs. However, vitiligo lesions in all sites were associated with significantly reduced levels of HRQL. To the best of our knowledge the location of CL lesions and impact on the HRQL has not yet been studied. In this study we aim to establish whether the body location of the CL lesions affects the HRQL of LCL patients in Suriname. We hypothesized that LCL located on the head/face would be associated with lower HRQL as compared to other locations on the body. While LCL located on the limbs would affect mobility and daily activities more as compared to LCL on the trunk and head/face.

## Methods

### Patients

The present study is conducted in the framework of the PELESU project (Clinical, Parasitological and Pharmaco-Economic evaluation of 3 days versus 7 days pentamidine isethionate regimen for cutaneous leishmaniasis in Suriname, Trial ID: NTR 2076; reviewed and approved by the Ethics Committee of the Ministry of Health in Suriname, file number VG006–2009) as part of the *Leishmania* Research Program in Suriname, an integrated research program comprising 3 different study scopes: clinical, biological, and medical-anthropological. Within the medical-anthropology component, disease perceptions and treatment seeking behavior of LCL patients within different cultural, socio-economic, occupational, environmental and geographical contexts was studied [4]. All enrolled LCL cases were patients from the outpatient clinic of the Dermatology Service in Paramaribo, Suriname and were recruited between January 2010 and May 2013, and data analysis for the anthropological component was concluded mid-2015.

Clinical details, age, sex, area of residence, perceived area of LCL acquisition, occupation, duration and location of the lesions, number, size, and treatment method, were recorded in case record files. When a patient had more than one lesion, the largest was considered the study lesion. Written informed consent was obtained from all participants. Standard questionnaires (see below) were used to assess HRQL and disease burden. The questionnaires were filled out by the participants before the initiation of treatment.

Furthermore, among a subset of patient’s various other aspects relating to illness, experience and aspects of stigma were studied through qualitative interviews. For this study, patients with different cultural, socio-economic, occupational, environmental and geographical contexts were purposively recruited.

### Questionnaires

#### Skindex-29

For HRQL assessment the skindex-29 questionnaires were used. HRQL reflects the patient’s evaluation of the impact of a disease and treatment on his/her physical, psychological, and social functioning and well-being) [11,12]. The skindex-29 is a three-dimensional, dermatology-specific HRQL questionnaire. Twenty-nine questions are combined to form three domains: symptoms, emotions and functioning. The domain scores and overall score are expressed on a 100-point scale, with higher scores indicating lower levels of HRQL [12,13,14,15,16,17].

#### EQ-5D-3L

The EQ-5D is a widely used measure of generic HRQL containing five domains (mobility, self-care, daily activities, pain/discomfort, and anxiety/depression). Each dimension has three response levels (no problems, some problems, extreme problems). Additionally, the EQ5D records the respondent’s self-rated health on a vertical, visual analogue scale (VAS) ranging from 0 “worst imaginable health state” to 100 “best imaginable health state” [18].

#### Illness experience

In addition to Skindex-29 and EQ-5D-3L, a semi-structured questionnaire containing 9 topics and related to those a number of open-ended questions (more or less than twenty nine depending on the answers provided by the patient) was developed to obtain more qualitative and contextualizing data. The questionnaire is included in S1. Among these questions, six (subdivided in variable groups of sub-questions) were specifically focused on dimensions of stigma [19], and how LCL patients experienced the illness in their day-to-day lives. The exploratory inquiries were partly grafted on the stigma assessment guidelines, developed by the International Federation of Anti-leprosy Associations (ILEP) and the Netherlands Leprosy Relief (NLR) [20]. This questionnaire was administered during a face-to-face interview among a subset of CL patients who took part in both the clinical and the (larger) anthropological study (46 in total). All 46 patients were interviewed at the Dermatology Service. Patients expected to be able to provide rich information on specific issues, e.g. with lesions on varying body parts, were invited for follow-up in-depth interviews. All interviews were conducted by the second lead author of this article (SR), a medical anthropologist. All answers were either written down on the form (by the researcher or research assistants), or in some cases first audio-recorded and then transcribed. The information obtained from the qualitative inquiries, in this article, are used to complement the clinical (quantitative) findings providing a more in depth emic view on how the illness is perceived and experienced by the LCL patients, specifically in relation to the duration, location, number and size of the leishmaniasis lesions on their bodies.

### Statistical analysis

Participants were allocated to one of four groups according to the location of leishmaniasis lesions on one of the body regions in the following hierarchy: 1) face/head, 2) upper limbs, 3) lower limbs and 4) trunk. If more than one location was involved, the presence of lesions in the lowest rank number determined the final group allocation. This hierarchy was based on our presumption that lesions in the face would have the greatest effect on social interaction and cause most stigma and that lesion on the upper limbs would have a greater impact on daily activity than lesions on the lower limbs.

Skindex-29 and EQ-5D-3L data was entered in epidata and qualitative information about stigma/ illness experience was entered into the computer as Word documents. Statistical analysis was conducted using SPSS version 19 (IBM).

The mean scores on the skindex-29 subscales and the EQ5D/VAS were compared among the four groups using general linear models. If the overall effect for location of the lesions was found to be statistically significant, post-hoc tests were conducted to examine which groups were statistically significantly different from each other with respect to their HRQL. Two-sided p-values of <0.05 were considered to indicate statistical significance.

The responses on the EQ5Q dimensions were dichotomized into patients reporting no problems versus patients reporting some or extreme problems. We compared the likelihood of reporting problems percentage of patients reporting no problems, some problems and extreme problems on the five EQ5D dimensions among the four groups using logistic regression analysis. Patients with lesions in the head/face served as reference group. Results are expressed as odds ratios.

The analyses with age and gender were repeated and included as covariates in the general linear models and in the logistic regression analyses to examine the effect of these covariates on the results.

A Spearman’s rank order correlation coefficient was calculated between the patients’ total number of lesions and the skindex-29 subscales and EQ5D dimensions. Correlation coefficients ≤0.10, 0.25 and 0.40 were considered small, medium and large effects, respectively [22].

The qualitative data obtained from the questionnaires concerning illness experience were coded and entered into a thematic framework of SPSS, version 19 (IBM), and were, after categorization and labeling, thematically entered into different groups of Excel spreadsheets. Thematic content analysis helped to analyze the data and extract relevant relationships between the research findings.

## Results

### Study population

The baseline characteristics of the 163 patients enrolled are presented in Table 1.

**Table 1:** Baseline characteristics of the study population (N = 163) of patients with localized cutaneous leishmaniasis in Suriname.

Characteristics	N	%
**Male gender**	150	92.0
**Age in years**		
Mean ±SD	32 ± 10.4	
Range	16–75	
Ethnic group^*^		
Maroon	69	42.3
Hindustani	31	19.0
Javanese	23	14.1
Creole	11	6.8
Amerindian	9	5.5
Other	20	12.3
**Number of lesions per subject**		
1	84	51.5
2	29	17.8
≥3	50	30.7
**Location of study lesions**		
Head and face	23	14.1
Upper limbs	40	24.5
Lower limbs	69	42.3
Trunk	31	19.1

* Ethnic group: Maroon—descendants of runaway slaves, traditionally living in the interior / Hindustani -descendants of British-Indian immigrants / Javanese—descendants of Dutch Indian immigrants / Creole—descendants from slaves, traditionally living in urbanized areas /Amerindians—indigenous population.

### Health-related quality of life (HRQL)

Of the patients 51.5% (84/163) had one lesion, 17.8% (29/163) had two lesions and 30.7% (50/163) had three or more lesions. All 163 patients were divided in the 4 groups according to the location of the study lesion in the following order: head/face (23), upper limbs (40), lower limbs (69) and trunk (31).

The mean skindex-29 scores of the four groups are presented in Table 2. We found a statistically significant effect for location of the lesions for the skindex-29 subscales symptoms (*p* = 0.034). Post hoc tests revealed that patients with lesions on the head/face had significantly lower symptoms scores, indicating a better HRQL, than patients with lesions on the lower limbs (*p* = 0.03). Moreover, patients with lesions on the upper limbs also had significantly lower symptom scores, indicating better HRQL, than patients with lesions on the lower limbs (mean score 33.5 versus 40.6, *p* = 0.01). On the emotions scores, lesions on the upper limbs caused marginally significant lower scores compared to lesions in the face/head (p = 0.05), whereas no significant differences were found as far as lesions on the lower limbs or trunk compared to face/head located lesions.

**Table 2:** Skindex-29 Mean scores for 4 locations of leishmaniasis lesions and compared to localization head/face.

Variables	Skindex								EQ5D	
	Symptoms mean (se)	p-value^2^	Emotions mean (se)	p-value^2^	Functioning mean (se)	p-value^2^	Total score	p-value^2^	EQ5D VAS	p-value^2^
**Location of lesions**										
Head/face^1^ n = 23	35.5 (3.3)	0.046	39.7 (3.7)	0.11	37.8 (3.7)	0.49	37.9 (3.2)	0.35	75.7 (3.1)	0.99
Upper limbs n = 40	35.4.0 (2.6)		30.8 (2.9)		32.2 (2.9)		32.5 (2.6)		76.1 (2.4)	
Lower limbs n = 69	42.0 (2.2)*		32.6 (2.5)		36.1 (2.6)		36.3 (2.2)		75.5 (2.1)	
Trunk n = 31	39.8 (3.1)		35.8 (3.6)		36.1 (3.6)		36.9 (3.1)		75.6 (2.9)	
**Gender**										
Male^1^ n = 150	37.0 (1.1)	0.32	28.0 (1.3)	0.003	27.9 (1.3)	0.001	29.9 (1.1)	0.002	80.6 (1.1)	0.008
Female n = 13	40.9 (3.7)		41.5 (4.3)*		43.2 (4.3)*		41.9 (3.7)*		70.8 (3.6)*	
**Age (quartiles)**										
16–23 years^1^ n = 40	36.8 (2.1)	0.52	28.7 (2.4)	0.054	37.4 (3.2)	0.026	36.6 (2.7)	0.051	76.4 (2.6)	0.16
24–31 years n = 42	39.9 (2.1)		32.6 (2.4)		39.9 (3.2)		40.1 (2.7)		72.6 (2.6)	
32–38 years n = 43	35.9 (2.1)		24.8 (2.3)		29.8 (2.9)*		32.0 (2.5)		78.9 (2.4)	
39–75 years n = 38	36.6 (2.2)		26.3 (2.4)		35.3 (3.2)		34.9 (2.7)		75.0 (2.6)	

Se = standard error, 1 = reference category

* = statistically significant difference from reference category on post-hoc test, 2 = from multivariate general linear model

Patients with lesions on the lower limbs were significantly more likely to report problems on the EQ-5D dimensions mobility, self- care, and pain discomfort than patients with lesions on the head/face (Table 3). Patients with lesions on the lower limbs were also more likely to report problems than patients with lesions on the upper limbs on daily activities (OR 2.6, *p* = 0.02) and on self- care (OR 3.4, *p* = 0.004). We observed no statistically significant difference in mean and median EQ VAS scores among the 4 groups with the different localization of the lesion (Table 4).

**Table 3:** EQ-5D for the 4 localizations of cutaneous leishmaniasis lesions, patients reporting problems and compared to localization head/face, by age and gender.

Variables	EQ5D									
	Mobility OR (95% CI)	p-value^1^	Self-care OR (95% CI)	p-value^1^	Daily activities OR (95% CI)	p-value^1^	Pain discomfort OR (95% CI)	p-value^1^	Anxiety/depression OR (95% CI)	p-value^1^
**Location of lesions**										
Head/face n = 23	reference		reference		reference		reference		reference	
Upper limbs n = 40	-	0.98	0.9 (0.3–3.3)	0.99	0.8 (0.3–2.5)	0.77	2.4 (0.8–7.8)	0.13	1.9 (0.6–5.6)	0.25
Lower limbs n = 69	12.5 (1.6–100.8)	0.012	3.5 (1.2–10)	0.02	2.3 (0.8–6.1)	0.09	5.6 (1.9–16.6)	0.002	2.1 (0.8–5.7)	0.14
Trunk n = 31	3.05 (0.3–30.7)	0.34	1.7 (0.5–5.8)	0.38	1.2 (0.4–3.7)	0.73	3.2 (0.9–10.8)	0.06	1.8 (0.6–5.6)	0.33
**Gender**										
Male n = 150	reference		reference		reference		reference		reference	
Female n = 13	0.7 (0.1–4.1)	0.72	1.8 (0.5–6.1)	0.36	1.1 (0.3–3.3)	0.98	5.2 (1.0–25.9)	0.049	3.7 (0.9–14.8)	0.064
**Age (quartiles)**										
16–23 years n = 40	reference		reference		reference		reference		reference	
24–31 years n = 42	0.5 (0.1–1.7)	0.25	0.9 (0.4–2.4)	0.95	1.1 (0.4–2.7)	0.86	1.5 (0.6–3.9)	0.37	0.9 (0.4–2.3)	0.86
32–38 years n = 43	1.2 (0.3–4.1)	0.81	0.8 (0.3–2.0)	0.65	1.2 (0.5–3.0)	0.66	1.1 (0.4–2.7)	0.90	0.6 (0.3–1.6)	0.31
39–75 years n = 38	1.2 (0.4–4.1)	0.75	0.5 (0.2–1.3)	0.17	1.3 (0.5–3.3)	0.58	0.7 (0.3–1.9)	0.49	1.7 (0.7–4.4)	0.25

OR = odds ratio, CI = confidence interval, 1 = from multivariate logistic regression model.

**Table 4:** EQ VAS score of 4 locations of cutaneous leishmaniasis lesions compared to localization head/face.

EQ5D VAS	Head/face	Upper limbs	Lower limbs	Trunk
	N = 23	N = 40	N = 69	N = 31
Mean (se)	79.9 (2.6)	79.9 (2.0)	78.9 (1.5)	81.6 (2.3)
difference with head/face	-	0.0	-0.09	1.7
p-value post hoc test	-	0.99	0.76	0.62

The impact of location of the lesions on HRQL did not change when age and gender were considered as covariates in the model. However, we found that women had significantly higher scores, indicating lower HRQL, than males on the emotions (38.7 versus 27.3, *p* = 0.01), functioning (41.3 versus 27.9, *p* = 0.003), and the total skindex-29 scale (40.3 versus 29.9, *p* = 0.007). Women were more likely to report problems on the EQ-5D dimension pain/discomfort (OR 5.1, *p* = 0.05) and they had significantly lower scores, indicating a lower HRQL, on the EQ-5D/ VAS (71.3 versus 80.6, *p* = 0.01).

We found that a higher number of leishmaniasis lesions was significantly correlated with higher scores, indicating lower HRQL, on the skindex-29 scales emotions (r = 0.17, *P* = 0.03), functioning (r = 0.27, *P* = 0.001) and overall (r = 0.23, P = 0.003). Patients with multiple lesions had significantly higher scores, indicating more problems, on the EQ-5D dimension daily activities (r = 0.22, *P* = 0.006).

### Illness experience

The qualitative interviews, acquired from 46 patients, showed that overall, LCL patients felt bad they had contracted an illness like LCL, because of the “cruel”, and hard to cure character of the illness. Eighty-nine % (41/46) viewed LCL as a serious or very serious illness, because of the harsh biomedical treatment it required, and the vicious, ‘flesh-eating’ character of the illness. Eighty-five % (39/46) of the patients thought of LCL as a very dangerous illness because of possible health threats such as losing a finger, arm, or leg.

Six of 7 patients with lesions on the head and face, reported not to have experienced any negative reactions from their family members or others in their social environment (friends, colleagues, and people in the neighborhood) nor did the vast majority of patients with lesions on the lower, or upper limbs, or the trunk (32 out of 39 patients). They commented to be treated “normally,” or “no different than usual”. Most of the patients said that people, especially the family, were taking care of them whenever needed. Six patients (five males and one female), all with lesions on their upper and lower limbs, had difficulties with their illness in terms of appearance; they felt ashamed for the lesions, and commented “they [the lesions] did not look good on the body.” These few patients also reported on being avoided by others because of their illness. In anticipation of negative remarks, or to avoid uncomfortable situations, some patients “kept distance” from others. The HRQL findings (Skindex-29 and EQ-5D questionnaires) that patients with lesions on the lower limbs were more likely to report problems than those with lesions on their head/face is thus (partially) supported by the qualitative findings. Apart from these negative experiences, however, life continued for patients “as usual.” From the 46 patients only two worried about scars; all others were only concerned about getting cured.

Twenty-five patients said they were worried about other aspects of their condition. Sixty % of these patients (15) felt mostly so because of the expected long treatment duration, uncertainty about the efficacy of the treatment, and the aggressiveness of their illness. Inquiries showed that almost all patients (42) had been discussing their condition in their social environment (with family members, colleagues and others) prior to visiting the Dermatology Service; amongst other matters, information about the treatment duration was ‘common’ knowledge. From the 46 patients, 20% (9) feared the injections, and were anxious about its painfulness, and the number of injections they had to receive before the lesions completely healed. Two patients were worried because of the lack of preventive medicine for the illness.

## Discussion

This study addresses how the body location of leishmaniasis lesions impacts on Health-related quality of life (HRQL). Although data collection for this study was initiated almost 10 years ago and data analysis completed 5 years ago, the presented information is still very valuable. To our knowledge, this is the first and to date only research to study the body location of lesions and the impact on the quality of life of leishmaniasis patients in Suriname using the Skindex-29 and EQ-5D/VAS to assess HRQL in patients with localised cutaneous leishmaniasis (LCL). Furthermore, we acquired qualitative data to complement the quantitative HRQL data, providing a more in-depth emic view on how the illness is perceived and experienced by the LCL patients. As such the presented work is valuable to the Surinamese health authorities and the Surinamese community in particular, but unique and relevant for all other colleagues working on CL in different parts of the world. Studies assessing quality of life and stigma associated with leishmaniasis are few, particularly in the Americas; and the current findings add to our knowledge (gap) and literature.

The main findings of this study are that lesions located on the lower limbs had more HRQL impact compared to the other body locations. Significant differences were seen in Skindex-29 symptoms category comparing lower limbs to head/face. In the EQ-5D dimensions self-care, mobility and pain/discomfort participants in the lower limb group were significantly more likely to report problems than those in the head/face group. Compared to the upper limb group the lower limb group also was more likely to report problems on self-care and daily activities. Women had lower HRQL than men; in the skindex-29 categories emotions, functioning and overall, they had significantly higher scores, they were more likely to report problems in the EQ-5D dimension pain/discomfort and the EQ VAS score was significant lower. A higher number of lesions were correlated with a lower HRQL on skindex-29 categories emotions, functioning and overall scores and the EQ-5D dimension daily activities. Although a higher number of lesions were significantly associated with a lower HRQL outcome on the skindex-29 scale, especially in the emotional and functioning dimensions, the location of multiple lesions did not significantly affect these dimensions.

In general, LCL patients were not stigmatized because of their illness [23]. In this study the location of the lesions on the body, especially those in the face/head, did not seem to contribute to negative attitudes of the social environment towards LCL patients. Multiple lesions, nor lesion size were associated with enacted stigma. In this study, negative reactions due to CL are confined to a small group of patients who endured more severe forms of CL. Although some patients felt ashamed about their appearance, only two out of 46 patients were concerned about scars.

The impact on the impairment of HRQL has been studied in skin diseases, i.e. vitiligo, psoriasis and atopic dermatitis [11,21]. Only a few studies report the impact of leishmaniasis lesions on quality of life. These have indicated that CL can have a great psychological and social impact on the quality of life of patients due to disfiguring scar formation and mutilation leading to social stigmatization [5,6,7]. A large decrement in quality of life in patients with active CL lesions has been described for Sanliurfa, Turkey. In this study Yanik et al. used different criteria to assess the HRQL of 99 patients, 33 with active CL lesions, 33 with CL scars and 33 controls [6]. A pilot study performed in Belo Horizonte, Brazil reported a negative impact of CL on patients quality of life, after using the Dermatology Life Quality Index (DLQI) to assess the HRQL of 20 patients with CL [9]. Vares *et al*. studied HRQL in 124 CL patients in Iran using the DLQI as instrument and confirmed the impairment in HRQL [8].

The relationship between distribution of skin diseases and subsequent impairment of HRQL has recently been studied in vitiligo. It was found that the localization of vitiligo lesions determines the magnitude of different aspects of quality of life [11]. As far as we know we here present the first data on the relation of body localization of CL on the HRQL. As CL lesions occur predominantly on exposed areas of the body and studies mention social stigmatization of CL patients with active lesions and scars on the face [5,6,7], we expected to find more impact of CL on the head/face on HRQL than CL in other regions like upper limbs, lower limbs and the trunk. Although lesions located in the face did lead to a higher score, indicating a worse HRQL, in the emotions category of the skindex-29 instrument, this did not lead to a significant difference with the other body location groups. In contrast, we found a larger impact on HRQL of CL lesions on the lower limbs. These lesions hindered mobility, self-care and daily activities (work, hobbies) due to pain and discomfort more, than lesions on other parts of the body. Although CL usually heal with scar formation, lesions in Surinamese participants did not seem to lead to stigmatization and lesions on the head/face did not appear to have more HRQL impact compared to lesion on other body locations.

In our study, women have lower HRQL than men; as measured by both the skindex-29 and EQ-5D. In Suriname LCL is associated with visiting the interior (rain forest) for work or leisure related reasons [4]. As a result, LCL is seen more frequently in men than women (in our study 92,0% of the participants were male). Lesions were located more often on the limbs (upper–and lower) than on the face. In contrast to the body distribution found in our study, in the reports coming from Afghanistan and Pakistan lesions tend to be located more often on the face, and women and children were more often affected than men [7]. Moreover, the extent, and size of the lesions is possibly larger in the Middle Eastern setting. This could possibly explain why stigma and isolation from society is more often mentioned among CL cases in these Old-World CL endemic areas. The higher frequency of facial CL lesions occurring in the Middle East may be due to the local costume habit with only the face and parts of hands and feet uncovered and most of the body including the limbs covered, and thus protected from the vector of CL, the sand flies. In contrast, in a humid tropical climate as in Suriname, the population tends to have more body parts uncovered, especially the upper- and lower limbs exposing those body regions to sand flies.

Strengths of this study are its prospective design, the number of subjects and the use of validated questionnaires for HRQL, i.e. the Skindex-29 and EQ-5D/VAS. The Skindex-29 and EQ-5D/VAS are well-established approaches for the evaluation of HRQL in dermatological diseases [13]. A limitation of our study is that we do not differentiate between lesions on the face or scalp and other body locations (trunk, arm, or leg). We assume that a (large-sized) lesion in the face (which is rare in the dominant type of CL in Suriname) would have led to enacted or internalized stigma as in Afghanistan and Pakistan. A further limitation of the study is that we did not take into account the actual size of the leishmaniasis lesion. Most study participants only had one lesion, and in case more lesions were present, the largest lesion was considered to be study lesion. However, in how far lesion size affected quality of life and stigma was in the current study not further assessed.

In conclusion, in Suriname LCL lesions located on the lower limbs are associated with a lower quality of life compared to lesions on the head/face, trunk and upper limbs. In contrast to earlier reports from CL endemic areas in the Middle East, stigma related to CL seems to be virtually absent in Suriname. It is unclear whether these regional differences in HRQL and stigma due to CL are caused by culture-specific factors or a consequence of the clinical type of CL, which in Suriname rarely causes facial disfiguration.

## Supporting information

S1 File Semi-structured questionnaire for CL patients at the Dermatology Service in Paramaribo. (DOCX)

## References

[1] Burza S, Croft SL, Boeleart M; Leishmaniasis. Lancet 2018; 392:951–970. 10.1016/S0140-6736(18)31204-2 30126638

[2] World Health Organization (2010) Control of Leishmaniasis: report of the meeting of the WHO expert committee on the control of leishmaniasis. Geneva: World Health Organization 949

[3] Van der Meide WF, Jensema AJ, Akrum RA, Sabajo LOA, Lai A Fat RFM, Lambregts L, Schallig HDFH, van der Paardt M, Faber WR; Epidemiology of Cutaneous Leishmaniasis in Suriname: A Study Performed in 2006. Am. J. Trop. Med. Hyg., 79(2), 2008, 192–197. 18689623

[4] Ramdas S; Cruel disease, cruel medicine: Self-treatment of cutaneous leishmaniasis with harmful chemical substances in Suriname. Social Science and Medicine 2012; 75: 1097–1105. 10.1016/j.socscimed.2012.04.038 22704264

[5] Reithinger R, Aadil K, Kolaczinski J, Mohsen M, Hami S; 2005 Social impact of leishmaniasis, Afghanistan. Emerg Infect Dis 11:634–636. 10.3201/eid1104.040945 15834984 PMC3320322

[6] Yanik M, Gurel MS, Simsek Z, Kati M; 2004 The psychological impact of cutaneous leishmaniasis. Clin Exp Dermatol 29:464–467. 10.1111/j.1365-2230.2004.01605.x 15347324

[7] Kassi M, Kassi M, Afghan AK, Rehman R, Kasi PM; Marring leishmaniasis: the stigmatization and the impact of cutaneous leishmaniasis in Pakistan and Afghanistan.PLoSNegl Trop Dis. 2008; 2(10):e259 10.1371/journal.pntd.0000259 18958168 PMC2569210

[8] Vares B, Mohseni M, Heshmatkhah A, Farjzadeh S, Safizadeh H, Shamsi-Meymandi S, Rahnama Z, Reghabatpour L, Fathi O; Quality of life in patients with cutaneous leishmaniasis. Arch Iran Med. 2013 Aug; 16(8):474–7. doi: 013168/AIm.008 23906253

[9] Afghanistan: No medicine for leishmaniasis in Badakhshan Province. IRIN news-United Nations Office for the Coordination of Humanitarian Affairs. Available at: www.irinnews.org/report/78674/afghanistan-no-medicine-for-leishmaniasis-in-badakhshan-province

[10] De Castro Toledo AC, da Silva RE, Carmo RF, Amaral TA, ProfetaLuz ZM, Rabello A; Assessment of the quality of life of patients with cutaneous leishmaniasis in Belo Horizonte, Brazil, 2009–2010. A pilot study. Trans R Soc trop med Hyg 2013; 107:335–336. 10.1093/trstmh/trt021 23474473

[11] Silverberg JI, Silverberg NB; Association between vitiligo extend and distribution and quality of life impairment. JAMA Dermatol. 2013 2;149(2):159–64. 10.1001/jamadermatol.2013.927 23560296

[12] Guyatt GH, Feeny DH, Patrick DL; (1993) Measuring health- related quality of life. Ann Intern Med 118:622–9 10.7326/0003-4819-118-8-199304150-00009 8452328

[13] Guyatt GH, Osaba D, Wu W et al; (2002) Methods to explain the clinical significance of health status measures. Mayo ClinProc 77:371–83 10.4065/77.4.371 11936935

[14] Chren MM, Lasek RJ, Quin LM et al; (1996) Skindex, a quality-of-life measure for patients with skin disease: reliability, validity, and responsiveness. J Invest Dermatol 107:707–13 10.1111/1523-1747.ep12365600 8875954

[15] Chren MM, Lasek RJ, Flocke SA et al; (1997a) Improved discriminative and evaluative capability of a refined version of Skindex, a quality-of-life instrument for patients with skin disease. Arch Dermatol 133:1433–40 9371029

[16] Prinsen CA, Lindeboom R, Sprangers MA, et al; (2010) Health related quality of life assessment in dermatology: interpretation of Skindex-29 scores using patient-based anchors. J Invest Dermatol 130:1318–22 10.1038/jid.2009.404 20032989

[17] Prinsen CA, Lindeboom R, Korte JD; (2011) Interpretation of Skindex-29 scores: cutoffs for mild, moderate, and severe impairment of health-related quality of life. J Invest Dermatol 131:1945–7 10.1038/jid.2011.138 21593773

[18] EuroQol Group. (1990) Euro-Qol a new facility for the measurement of health related quality of life. Health Policy 16:199–208 10.1016/0168-8510(90)90421-9 10109801

[19] Jones E, Farina A, Hastorf A, Markus H, Miller D, Scott R; 1984 Social stigma: the psychology of marked relationships. New York: Freeman and Company.

[20] International Federation of Anti-leprosy Associations (ILEP) & the Netherlands Leprosy Relief (NLR). 2011 Guidelines to reduce stigma. London/Amsterdam: ILEP & NLR.

[21] Badia X, Mascaró JM, Lozano R; Cavide Research Group; Measuring health-related quality of life in patients with mild to moderate eczema and psoriasis: clinical validity, reliability and sensitivity to change of the DLQI. Br J Dermatol. 1999; 141 (4): 698–702 10.1046/j.1365-2133.1999.03112.x 10583119

[22] Cohen J; Statistical power analysis for the behavioural sciences. Mahwah, NJ: Erlbaum; 1988.

[23] Ramdas S, Van der Geest S, Schallig HD; Nuancing stigma through ethnography: the case of cutaneous leishmaniasis in Suriname. Social Science & Medicine. 2016; 151:139–46. 10.1016/j.socscimed.2015.12.044 26802370

