# Supplementary material for: Body location of “New World” cutaneous leishmaniasis lesions and its impact on the quality of life of patients in Suriname
Source: PLoS Negl Trop Dis. 2020 Oct 23;14(10):e0008759. doi: 10.1371/journal.pntd.0008759 (PMC7641459; doi:10.1371/journal.pntd.0008759)
Supplement: S1 File — (DOCX) [file pntd.0008759.s001.docx]

S1 File

**Semi-structured questionnaire for CL patients at the Dermatology Service in Paramaribo**

Instruction: Briefly introduce purpose of the interview. Emphasize that all information will be confidential. There are no wrong answers. If recording is made, explain and ask for permission. Check if informed consent has been taken.

1. Socio-demographic characteristics

* Age

* Sex

* Ethnicity

* Education

* Living area

* Working area

1. Knowledge of illness (aetiology)

* How do you call this disease? (Vernacular name)

* What, according to you, caused this disease?

* What do you know (or have heard) about this disease?

1. History of illness, steps in treatment seeking

* How long ago did you discover the sore(s)?

* What did you do after you discovered it (or them)? (Describe)

1. Self-treatment (if applicable)

* What did you treat your sore(s) with?

* Why with those medicines?

* On whose advice?

* What other types of medicines did you hear of (in the treatment of this disease)?

* You tried to self-medicate, why did you do so?

* Why only now did you go to the doctor?

1. Stigma and illness perceptions

* How did you feel when you discovered the sores?

* How do/did you feel about having sores on your body?

* Why did/do you feel that way?

* How do those in your social environment (family, neighbours, friends, co-workers, students and others) react when they see the sores? (Probing)

* Do you think you are treated differently because of the sores?

* If yes, how, in which way? If no, why do you think so?

* Do you think the disease is serious? (If yes how serious?)

* Do you think this disease is contagious? (If yes, how, in which way?)

* Do you think this disease is dangerous? (If yes, why?)

* Do you think this disease is lethal? (If yes, why?)

1. Dietary or other behavioural changes

* Did you change anything in your food intake since you are infected with this disease?

* Are there certain (other) things you do or avoid because of the sores? (Probing)

1. Accessibility of biomedical care

* Was it easy or difficult to get to this clinic? (Explain)

1. Prevention

* How do you think you can prevent this disease?

1. Patients’ concerns and fears

* What is your biggest concern related to this disease?

* What is your biggest fear related to this disease?

1. Choice of medication

* If you had the choice between using bush medicines or (biomedical) pills, ointments, powder or injections (provided by the Dermatology Service), what would you choose and why?

1. Closing remarks

*Is there anything else you want to add, share or ask?

Instruction: Thank participant for providing the answers and the time. If patient is further being examined, accompany her/him to consultation room. Otherwise ensure that follow-up appointment has been made.
